# Deep learning–based identification of visually similar foliar diseases in field-grown barley

**DOI:** 10.1186/s13007-026-01532-7

**Published:** 2026-04-18

**Authors:** Sofia Martello, Nikita Genze, Dominik G. Grimm

**Affiliations:** 1 Bioinformatics, Technical University of Munich, Campus Straubing for Biotechnology and Sustainability, 94315 Straubing, Germany; 2 Bioinformatics, Weihenstephan-Triesdorf University of Applied Sciences, 94315 Straubing, Germany; 3 TUM School of Computation, Information and Technology, Technical University of Munich, 85748 Garching, Germany

**Keywords:** Plant phenotyping, Semantic segmentation, Deep learning, Disease resistance, Barley, Computer vision

## Abstract

**Background:**

Accurate segmentation of foliar diseases under field conditions is essential for large-scale phenotyping, as breeding programs rely on reliable severity estimates to identify genotypes with improved resistance. However, most deep learning approaches have been developed as pathogen-specific models, which limits scalability in field-grown barley where multiple diseases naturally co-occur and exhibit substantial visual similarity.

**Results:**

We evaluated whether a multiclass segmentation model can simultaneously detect and distinguish two fungal diseases of barley, *Puccinia hordei* and *Ramularia collo-cygni*, and compared its performance with two disease-specific binary models. Using 336 high-resolution leaf scans collected in the field with naturally occurring co-infections, the multiclass model achieved higher Dice scores for brown rust (0.59 vs 0.40; +47.5% relative improvement) and ramularia (0.60 vs 0.53; +13.2% relative improvement). It also captured a greater proportion of individual lesions across both classes. At the genotype level, the model-predicted disease area percentages were highly consistent with those from ground truth annotations ($r > 0.99$).

**Conclusions:**

A unified multiclass framework can more effectively segment visually similar foliar diseases than separate binary models, while simplifying the computational workflow. This provides a scalable basis for automated resistance assessment within breeding pipelines. Code and data are publicly available at https://github.com/grimmlab/BarleyDiseaseSegmentation, with Mendeley Data dataset DOI 10.17632/4ny92p2r8f.1.

**Supplementary Information:**

The online version contains supplementary material available at 10.1186/s13007-026-01532-7.

## Introduction

Global agriculture faces a critical challenge: reducing its environmental footprint while simultaneously increasing food production, as emphasised by initiatives such as the European Farm to Fork Strategy [1, 2]. Improving the stability of yields in major crops, including cereals (ideally with fewer chemical inputs), has therefore become a key priority. Achieving yield stability also depends on developing cultivars resistant to foliar pathogens, which cause substantial annual losses. Barley (*Hordeum vulgare*), a central component for feed and malting, is highly susceptible to fungal diseases such as brown rust (*Puccinia hordei*) and ramularia leaf spot (*Ramularia collo-cygni*), which significantly impact its yield and quality [3–6]. Current management relies mostly on chemical fungicides, which contribute to environmental contamination and promote pathogen resistance [7]. For this reason, improving genetic resistance has become a central task of sustainable agriculture [8].

A critical approach to enhancing genetic resistance in plants is known as resistance breeding. This method involves cultivating a diverse range of plant genotypes, infecting them with a pathogen, and assessing their responses through phenotyping. Phenotyping is performed by experts who visually evaluate the plants and score the severity of the infection, which indicates the proportion of visibly diseased leaf area. After this assessment, the most promising resistant cultivars are selected for the next breeding cycle. This process continues until a suitable genotype is developed, which exhibits all the desired traits. Currently, the development of resistant cultivars is delayed by an ongoing phenotyping bottleneck. Expert visual scoring constitutes a significant limitation, despite being the predominant method for evaluating resistance. Although standardised [9–11], this approach is subjective and exposed to human error, including rater fatigue and inconsistency [12, 13]. These constraints hinder breeding, leading to the selection of suboptimal genotypes and introducing significant noise into phenotypic datasets. This complicates the discovery of genetic markers [14–16] and masks the heritable signal in QTL mapping studies [17]. High-throughput, automated phenotyping offers a promising approach to overcoming these issues by enabling objective, scalable quantification of disease.

In the context of plant disease assessment, deep learning-based semantic segmentation has emerged as a powerful tool [18]. By performing pixel-wise classification, segmentation models could directly quantify diseased leaf area, providing breeders with the severity metrics they need. Convolutional neural networks, particularly the U-Net architecture [19], have been successfully applied to segment diseases in various crops, including grapes, wheat, and maize [20–26]. A common limitation in prior work is the reliance on simplified conditions, such as controlled laboratory infections [27] or single-disease studies [21], which cannot adequately model the heterogeneous and co-occurring disease pressures found in the field. Despite its economic significance, image-based segmentation of barley’s major foliar diseases remains largely underexplored. Most research has concentrated on net blotch (*Pyrenophora teres*) [28, 29], while other diseases, such as brown rust and ramularia, have received considerably less attention. These diseases create small, necrotic lesions that are visibly similar and often co-occur on the same leaf. This co-infection presents challenges even for expert human identification, as the lesions can be difficult to distinguish. For automated systems that rely on structural or colour differences, this similarity complicates accurate diagnosis. Additionally, previous studies on related rusts in other crops have primarily utilised clean, laboratory-grown leaf images [27], highlighting a critical gap in understanding model performance with complex, field-derived imagery.

In this study, a high-resolution dataset of field-infected barley leaves was constructed to address this gap, including pixel-level annotations distinguishing brown rust, ramularia, and healthy tissue. This dataset, due to its field-grown nature and the presence of various fungal diseases, captures the real-world complexities of co-infection and varying symptom presentation. The segmentation task is particularly challenging due to the small size of lesions, their colour similarity to senescent tissue, and their irregular boundaries.

The central research objective of this study is to evaluate whether a single, multiclass deep learning model can effectively segment and distinguish visually similar barley leaf diseases (e.g., brown rust and ramularia) in complex, real-field images, and to compare its performance with that of dedicated binary models. For this purpose, a comparative performance evaluation was designed. The rationale for expecting a multiclass model to be effective is based on the task itself: when segmenting co-occurring and visibly similar diseases, a model that must distinguish all classes simultaneously might develop more robust representations than separate binary models, each of which could learn to identify generic symptomatic tissue without resolving inter-class ambiguity. This study, therefore, assesses whether this potential advantage translates into measurable performance improvements in a real-world setting, i.e., segmentation accuracy, lesion detection, and correlation of disease-area percentages at the genotype level.

## Materials and methods

The experimental pipeline, from data acquisition to model evaluation, is summarised in this section.

### Data collection and preprocessing

**Fig. 1:**
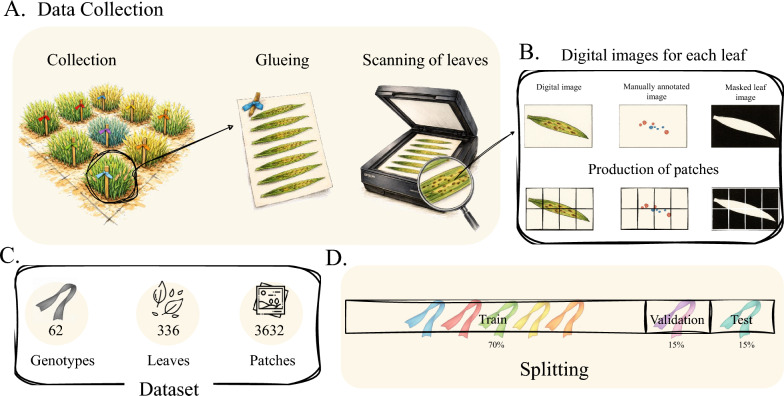
Overview of the data workflow. **A** Field-collected barley leaves (approximately eight per genotype) were flattened by glueing to avoid deformation and scanned at 800 dpi. **B** Each scan was manually annotated to label brown rust and ramularia lesions. A separate leaf mask was created to remove background pixels from subsequent calculations. Full-resolution images and masks were divided into $512\times 512 \text {px}^2$ patches for model training. **C** Summary of the resulting dataset: 62 genotypes, 336 scanned leaves, and 3632 extracted patches. **D** Genotype-level split of the dataset into training (70%), validation (15%), and test (15%) sets, ensuring that each subset contains distinct, non-overlapping genotypes

Winter and spring barley (*Hordeum vulgare*) genotypes were grown in field trials at three locations during the 2024–2025 seasons (Estrées Saint Denis, France; Irlbach and Paitzkofen, Germany; Supplementary Table S1). Flag leaves were sampled at the heading–flowering stages (Zadoks 50–69 [30]) from two populations: 238 winter barley genotypes susceptible to brown rust and 405 spring barley genotypes susceptible to ramularia. Field conditions produced natural multi-pathogen infections, yielding a broad severity gradient: 87% of leaves contained at least one disease, and 46% showed co-infection. To ensure biological replication, twelve leaves were collected per winter-barley genotype and four to five per spring-barley genotype. Leaves were mounted on A4 sheets and scanned at 800 dpi under standardised lighting (Fig. 1A). In addition to the annotated subset used for model development, an independent set of unlabelled leaves collected during the same field trials at Estrées-Saint-Denis, Irlbach, and Paitzkofen was included for population-level analysis. It includes 2,092 winter barley leaves representing 199 genotypes, 669 spring barley leaves collected in 2024 from 195 genotypes, and 882 spring barley leaves collected in 2025 from 161 genotypes. This independent dataset supports the evaluation of disease prevalence and patterns across winter and spring barley populations, including previously unseen genotypes. After that, ground truth annotations were generated manually at the pixel level on a subset of images due to the highly time-consuming nature of the process. An annotator, supported by industry barley breeders, used a Wacom tablet to delineate lesions, assigning distinct colours per pathogen to differentiate them during analysis. In total, 106,330 brown rust and 59,996 ramularia lesions were annotated across all annotated leaves. While meticulous, the annotations are not perfect. Subtle annotation errors at class boundaries and in areas of mixed symptoms introduced label noise, a common limitation in segmentation datasets and may affect performance metrics [31]. Preprocessing generated, for each leaf, an RGB image, a background mask, and a segmentation mask (Fig. 1B). CLAHE was applied in LAB space [32, 33] to enhance lesion visibility. Full-resolution scans were split into $512\times 512$ px patches for memory-efficient training (Fig. 1C). A genotype-level, stratified split (Fig. 1D) yielded 242 training leaves (43 genotypes), 35 validation leaves (9 genotypes), and 59 test leaves (10 genotypes), preventing genotype-specific artefacts from inflating performance [34]. As lesions occupy only a small fraction of pixels (Table 1), extensive augmentation was applied to improve model robustness (Supplementary Table S3).

**Table 1:** Leaf pixel class distribution across dataset splits

Class	Train (%)	Validation (%)	Test (%)
Healthy	97.47	96.43	98.14
Brown rust	1.05	0.91	1.09
Ramularia	1.48	2.66	0.75

Values represent the percentage of total pixels

This study is challenging because the two diseases are manifestly similar and often co-occur on the same leaf (Supplementary Figure S4). Quantitative analysis of the annotated lesions (Supplementary Note S2) further confirms that, while the diseases differ in colour and morphology, these characteristics overlap substantially, underscoring the difficulty of reliable discrimination. In addition, the dataset is dominated by healthy tissue pixels, and lesions typically occupy only a small fraction of the leaf area (Table 1). Lesion sizes are highly skewed for both diseases, with the vast majority of lesions very small, with a median lesion size of 40 px$^2$. Together, the visual similarity, overlapping lesion features, and predominance of small lesions define the complexity of the automated segmentation task.

### Model architecture and training

To assess the viability of a single multiclass model for simultaneous segmentation and disease discrimination, a U-Net architecture was employed [19]. This choice was based on its established efficacy in semantic segmentation, as demonstrated in recent plant phenotyping studies [35–37], and its architectural suitability. Specifically, U-Net’s skip connections facilitate the precise delineation of small, heterogeneous lesions by fusing high-level context with fine-grained spatial information [38]. Furthermore, the U-Net framework provides a controlled, computationally feasible baseline. This focused comparison is critical, as alternative approaches like transformer- or prompt-based foundation models typically require different training protocols, substantially greater resources, and are not directly comparable without extensive adaptation.

**Fig. 2:**
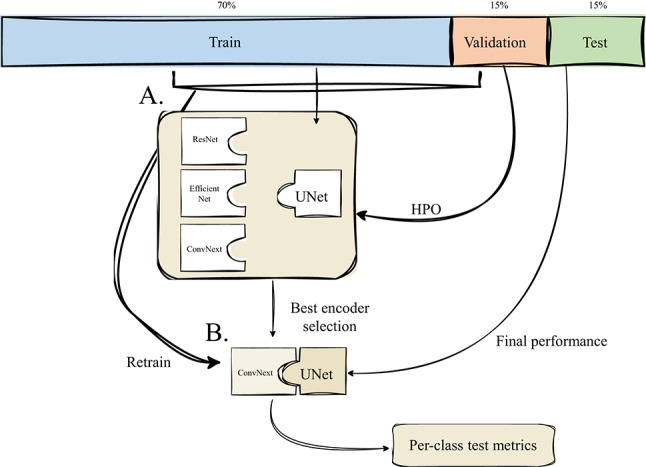
Model development and evaluation workflow. The dataset was split at the genotype level into training (70%), validation (15%) and test (15%) subsets. **A** Three U-Net models with different encoder backbones (ResNet34, EfficientNet-B2 and ConvNeXt-Tiny) were trained and optimised through hyperparameter optimisation (HPO) using the training and validation sets. **B** The encoder with the best validation performance was then selected and retrained from scratch on the merged training and validation set. The final model was evaluated on the held-out test genotypes, producing per-class performance metrics

The experimental design evaluated two complementary factors: task formulation and encoder architecture. Three formulations were tested: (i) a binary model for ramularia; (ii) a binary model for brown rust; and (iii) a multiclass model predicting background, ramularia, and brown rust simultaneously. To evaluate feature extraction strategies for this dataset, three ImageNet-pretrained encoders were integrated into the U-Net backbone (Fig. 2A), selected to represent key architectural paradigms while maintaining a focus on practical efficiency for potential field deployment. ResNet34 [39, 40] was chosen as a standard residual baseline, providing a well-understood trade-off between depth and computational cost compared to larger (e.g., ResNet50) or smaller variants. EfficientNet-B2 [40, 41] was selected from the EfficientNet family as a model that offers a favourable compromise between its parameter efficiency and representative capacity, avoiding the diminishing returns of larger models (e.g., B3-B7) for this task. Finally, ConvNeXt Tiny [40, 42] represents a transformer-inspired convolutional design. The ’Tiny’ variant was specifically chosen because it is the lightest model in the family, aligning with the efficiency objective and providing a direct counterpart to the other mid-sized encoders. Overall, this yielded nine model variants (3 tasks $\times$ 3 encoders). For each task formulation, the optimal encoder was selected based on validation performance.

Hyperparameter optimisation (HPO) was conducted for each encoder–task combination using a Tree-structured Parzen Estimator (TPE) sampler [43], which efficiently explores complex search spaces compared with, for example, random search. In HPO, the learning rate, dropout, and the weighting factor ($\lambda$) that balances the Dice and Focal loss terms were tuned. All models were trained with a weighted Dice–Focal loss, using inverse class frequencies to mitigate severe pixel imbalance. This formulation was selected because the Focal loss improves learning from under-represented classes, whereas the Dice loss is well-suited for small-object segmentation in imbalanced settings [44]. The loss formulation is provided in Supplementary Note S3.

Models were optimised with AdamW [45], a reduce-on-plateau scheduler, automatic mixed precision, gradient clipping, and early stopping. Full search ranges and augmentation settings are listed in Supplementary Tables S3–S4. After encoder selection, final models for each task were retrained on the combined training and validation sets (Fig. 2B) and evaluated on the held-out test set to obtain unbiased generalisation estimates. To evaluate the model’s utility for breeding, the focus was on the agreement between predicted and ground truth disease area percentages, as this continuous measurement is the direct input to the visual assessment scores used by breeders. The practical scoring system (Supplementary Table S5) maps these percentages to an ordinal scale; therefore, high accuracy in predicting the area percentage is a prerequisite for accurate visual scoring.

### Evaluation metrics

A suite of complementary metrics was used to assess performance across scales, from pixel-level segmentation quality to breeding-relevant utility. Unless otherwise stated, metrics were computed per leaf and averaged over the held-out test set (59 leaves from 10 genotypes). Genotype-level analyses were performed on genotype means. Mathematical definitions and additional rationale for all metrics are provided in Supplementary Note S1.

Pixel-level segmentation performance was quantified using the per-class Dice score and Intersection over Union (IoU), which are commonly used and comparatively robust under strong class imbalance [44]. To evaluate the ability to localise individual lesions, instance-level detection metrics (precision, recall and F1-score) were calculated at two IoU thresholds: a lenient threshold ($\tau =0.2$) to capture lesion localisation and a stricter threshold ($\tau =0.5$) to evaluate boundary accuracy [46].

For breeding applications, an important objective is to prioritise genotypes based on disease resistance. Therefore, the agreement between model-predicted and ground truth disease area percentages was assessed by computing the Pearson correlation coefficient (*r*) at the leaf level ($r_{\text {leaf}}$) and at the genotype level ($r_{\text {genotype}}$), where genotype values were calculated as means across leaves. The genotype-level correlation reflects how well model outputs preserve between-genotype differences in disease severity, which is directly relevant for selection decisions [9].

In addition to correlation, prediction errors at the genotype level can be measured using mean absolute error (MAE) and root mean square error (RMSE). Both metrics are expressed on a 0–100 % scale, which matches the range of disease severity area values. MAE indicates the average size of the difference between predicted and ground truth values, whereas RMSE gives more weight to larger differences. Together, these metrics provide an intuitive absolute measure of how closely the predictions match the ground truth disease area.

## Results

### Model development and encoder selection

To enable consistent, comparative evaluations, an encoder backbone was selected. Three candidate encoders were evaluated using Bayesian optimisation on the validation set across three segmentation task formulations. ConvNeXt Tiny achieved the highest validation mean Dice score across all tasks (Table 2) and was therefore used for all subsequent experiments. All reported improvements refer to relative percentage changes, computed as $(\Delta / \text {baseline}) \times 100$. Note that the mean Dice values (and the corresponding percentage improvements) are not directly comparable across task formulations [46]. Binary models average Dice scores across two classes (background and one disease), whereas the multiclass model averages across three classes (background and two diseases). Consequently, differences in mean Dice values between task formulations reflect, in part, the task-dependent definition of the metric, and comparisons should be restricted to values computed identically, i.e. within the same task formulation. Full results are provided in Supplementary Table S6.

**Table 2:** Validation performance improvement over the worst-performing encoder within each task

Encoder	Binary ramularia (%)	Binary brown rust (%)	Multiclass (%)
	Relative improvement	Relative improvement	Relative improvement
ResNet34	+1.57	+0.00	+2.66
EfficientNet	+0.00	+1.77	+0.00
ConvNeXtTiny	**+2.16**	**+3.67**	**+4.35**

Values report the improvement in mean Dice score (best per task in bold). All improvements are expressed as relative percentage changes, computed as $(\Delta / \text {baseline}) \times 100$. Mean Dice scores are computed over different numbers of classes (binary: 2, multiclass: 3) and are therefore not directly comparable across tasks

### Comparative performance of binary vs. multiclass segmentation

A per-class comparison showed that the multiclass model yields higher scores than the disease-specific binary models for both on the test set. As summarised in Table 3, the multiclass model achieved a Dice score of 0.59 for brown rust, corresponding to a 47% relative increase over the binary model (0.40). For ramularia, the multiclass model likewise improved performance, increasing Dice score from 0.53 to 0.60 (+13% relative improvement).

**Table 3:** Per-class segmentation metrics comparing binary and multiclass models on the test set

Task	Brown rust		Ramularia	
	Dice	IoU	Dice	IoU
Binary	0.40	0.27	0.53	0.37
Multiclass	**0**.**59**	**0**.**43**	**0**.**60**	**0**.**44**

Values in bold represent the higher score when comparing binary versus multiclass performance

**Fig. 3:**
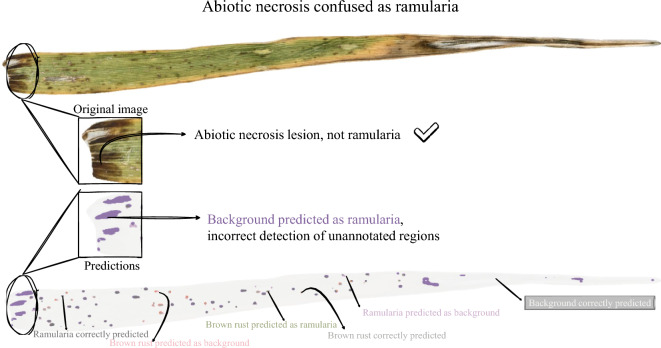
Example of the ground truth annotations being more accurate than the model predictions. In this case, the multiclass model confused necrosis with ramularia, whereas the ground truth showed no annotation in that area

**Fig. 4:**
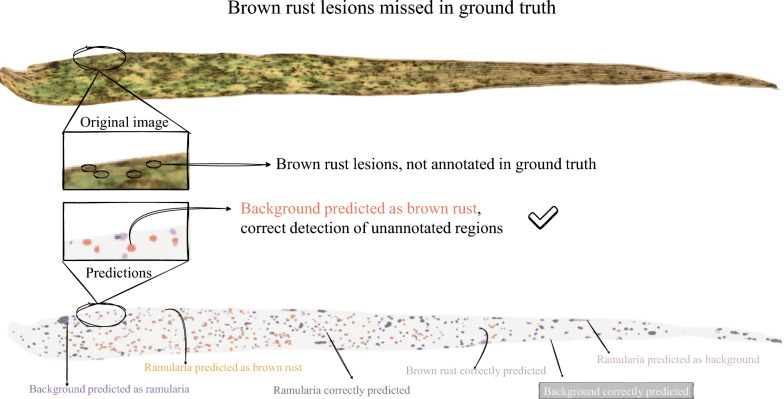
Example of the multiclass model predictions being more accurate than the ground truth. Here, brown rust lesions were not annotated in the ground truth, while the model predictions correctly identified them

The instance-level detection analysis further supported the multiclass model’s enhanced lesion-finding capability, though it also indicated that achieving accurate boundaries remained a key challenge (Table 4). Across both diseases, recall was consistently higher for the multiclass model. Moreover, the pronounced drop in performance from the lenient IoU thresholds ($\tau =0.2$) to the stricter threshold ($\tau =0.5$) suggests that the main limitation was precise boundary alignment rather than lesion localisation. At $\tau =0.2$, the multiclass model achieved high F1 scores (0.78 for brown rust and 0.77 for ramularia). In contrast, at $\tau =0.5$ performance decreased substantially (0.44 for brown rust and 0.41 for ramularia), reflecting the difficulty of delineating small, irregular lesions at pixel-level precision. Inspection of the output images highlighted cases where differences between predictions and annotations were attributable to ambiguity or omission. In Fig. 3, the model missclassified a large necrotic region as ramularia, whereas the ground truth contained no lesion annotation. Conversely, Fig. 4 shows an example where brown rust symptoms were not annotated in the ground truth but were detected by the model. Together, these examples suggest that discrepancies arise from not only model limitations (e.g., confusion with necrosis) but also from annotation uncertainty or omissions. These discrepancies can affect both quantitative metrics and qualitative interpretations.

**Table 4:** Instance-level detection metrics at different IoU thresholds

Disease	Task	IoU threshold 0.2			IoU threshold 0.5		
		F1	Prec.	Rec.	F1	Prec.	Rec.
Brown rust	Binary	0.47	0.40	0.75	0.25	0.22	0.40
	Multiclass	**0**.**78**	**0**.**75**	**0**.**85**	**0**.**44**	**0**.**42**	**0**.**48**
Ramularia	Binary	0.65	0.74	0.61	0.37	0.42	0.35
	Multiclass	**0**.**77**	0.73	**0**.**85**	**0**.**41**	0.40	**0**.**46**

The substantial drop in performance between F1@0.2 and 0.5 indicates that boundary precision, not lesion detection, is the primary segmentation challenge. Bold values indicate where the multiclass model outperformed the binary model on the given metric

### Genotype-level scoring accuracy

**Fig. 5:**
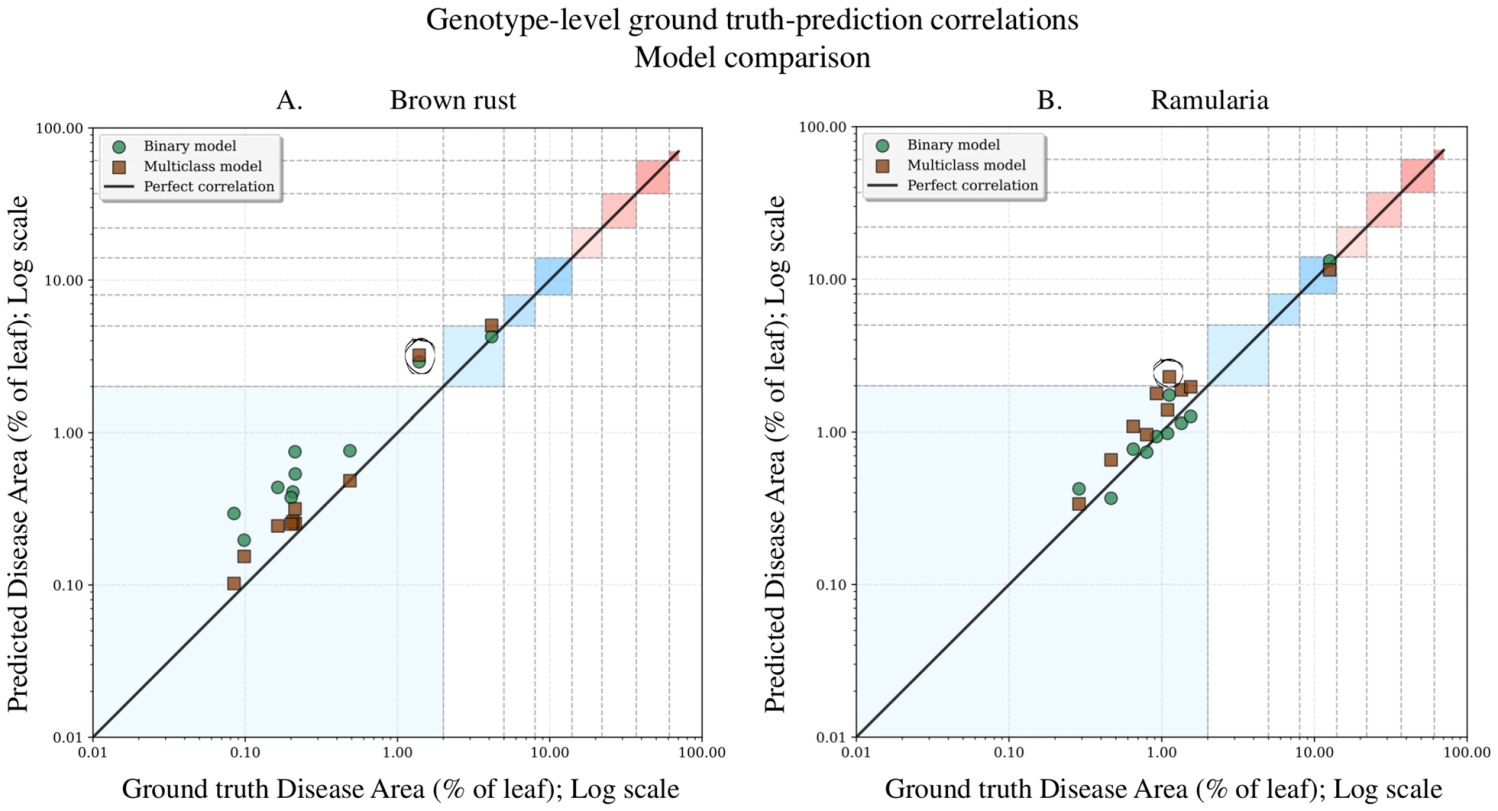
Genotype-level correlation between predicted and ground truth disease area percentages for brown rust (**A**) and ramularia (**B**). Points represent genotype means; the diagonal line indicates perfect correlation. The colored background grid shows the 9-point visual assessment scale for reference: Score 1 (0.0%), 2 (0.0–2.0%), 3 (2.0–5.0%), 4 (5.0–8.0%), 5 (8.0–14.0%), 6 (14.0–22.0%), 7 (22.0–37.0%), 8 (37.0–61.0%), 9 (61.0–100.0%). This visualisation allows simultaneous assessment of continuous correlation accuracy and alignment with breeding scoring protocols. Binary predictions: green circles; multiclass: brown squares

In addition to assessing pixel- and lesion-level performance, genotype-level disease severity was evaluated, a critical factor in breeding decisions. Pearson correlations between predicted and ground truth disease area percentages were computed across genotypes in the held-out test set (Fig. 5). Although two genotype means showed the largest deviation from the perfect correlation line (additionally circled in Fig. 5), both the binary and multiclass models achieved high genotype-level correlations (Table 5), indicating that between-genotype differences in disease severity were preserved reliably. While Fig. 5 provides an overview of genotype-level performance across all models, Supplementary Table S7 presents a detailed breakdown for each individual genotype. To complement the correlation analysis and provide a more comprehensive picture of agreement between predicted and ground truth disease severity, we also computed error-based metrics. Specifically, we calculated the mean absolute error (MAE) and root mean square error (RMSE) between the predicted disease severity (%) and ground truth visual assessments at the genotype level. As shown in Supplementary Table S10, the model achieved low error rates for both diseases, with MAE values of 0.31% for brown rust and 0.52% for ramularia, and RMSE values of 0.64% for both diseases. These low errors indicate that the model’s predictions closely match expert assessments, even within the narrow range of severity values observed in this study, further supporting the reliability of the approach for genotype-level phenotyping.

**Table 5:** Leaf-level and genotype-level correlation coefficients (Pearson’s r) between predicted and ground truth disease areas

Disease	Task	$r_{\text {leaf}}$	$r_{\text {genotype}}$
Brown rust	Binary	0.97	0.95
	Multiclass	0.98	0.96
Ramularia	Binary	0.97	0.99
	Multiclass	0.97	0.99

All correlation coefficients exceeded 0.95, with particularly strong genotype-level agreement for ramularia ($r_{\text {genotype}} \ge 0.99$), consistent with near-identical genotype ordering by disease severity. All correlations were statistically significant ($p < 0.001$, one-sided test with $H_0:\rho =0$ and $H_1:\rho >0$, Benjamini–Hochberg correction). These results suggest that automated segmentation is a scalable and consistent alternative to expert visual scoring for ranking genotypes in breeding trials.

### Validation of population-specific disease patterns

To evaluate whether the multiclass segmentation model preserves meaningful biological structures, we compared disease prevalence across in a winter barley population susceptible to brown rust and two spring barley populations selected for ramularia resistance and collected in 2024 and 2025. The model reproduced the expected population differences in the manually annotated test set (Supplementary Table S8). Winter barley exhibited significantly higher brown rust severity than the spring populations (5.04% vs. 3.22% for spring 2024 and 0.26% for spring 2025), which closely matched the ground truth (4.15% vs. 1.39% and 0.21%, respectively). Conversely, ramularia severity was higher in the spring 2024 population (11.52%, ground truth 12.59%) and lower in the spring 2025 population (1.54%, ground truth 1.01%), while winter barley remained low (0.34%, ground truth 0.29%). These differences were statistically significant (one-sided Mann–Whitney U test, $p < 0.05$ for Winter vs. Spring 2024 and $p < 0.001$ for Winter vs. Spring 2025) with large effect sizes ($r > 0.5$), indicating that the model captured the known population-level resistance contrasts.

At the genotype level, the model provides biologically plausible estimates of disease severity for both brown rust and ramularia, even when they co-occur on the same leaves. The close agreement between the model’s predictions and the ground truth demonstrates that disease expression can be reliably quantified within populations. This allows us to ranked genotypes according to their relative susceptibility to each disease.

These population-level patterns were also confirmed in a larger, unlabelled dataset of over 4,000 leaves from approximately 400 genotypes. This dataset included repeated collections of the same genotypes in 2024 and 2025 (Supplementary Table S9). In this dataset, winter barley exhibited significantly higher levels of brown rust (2.90%) than spring barely (1.27% in 2024 and 0.25% in 2025), while ramularia levels were highest in spring 2024 (23.00%) and lowest in spring 2025 (1.81%), consistent with the earlier collection time. Statistical tests confirmed that these contrasts were significant for winter vs spring 2024 (Mann–Whitney U test, $p < 0.001$ for both diseases).

Figure S6 illustrates the differences among populations and collection years. Winter barley genotypes cluster in the rust-dominated region (high brown rust and low ramularia). Spring 2024 genotypes occupy the ramularia-dominated region (low rust and high ramularia). Spring 2025 genotypes, which were collected earlier, demonstrate lower disease severity. These patterns demonstrate that the model captures genetic differences at population level and temporal variation in disease pressure.

## Discussion

### Performance and practical advantages of a unified model

This study demonstrated that a single multiclass model can segment and distinguish the similar foliar diseases brown rust and ramularia on field-grown barley. The unified model outperformed the disease-specific binary models for both classes, relatively improving per-class Dice score by 47% for brown rust and 13% for ramularia (Table 3). These gains are likely linked to the dataset’s characteristics, i.e., substantial overlap between symptoms and the predominance of very small lesions (Supplementary Figure S2), which introduce ambiguities that binary models, trained to separate one disease from background, may not resolve consistently. In contrast, jointly learning background, brown rust, and ramularia may encourage the model to exploit additional contextual information that supports discrimination between similar phenotypes. Multiclass labelling also reduces ambiguity in the background class, as pixels belonging to one disease are explicitly distinguished from the other, and might act as a form of implicit multi-task learning, regularising the network and enhancing generalisation. These factors might be helping the model capture global context, which would be especially beneficial when lesions are small or visually similar. However, if a disease is extremely rare or the class imbalance is severe, binary models may perform comparably well. Future experiments, such as feature attribution analyses, ablation studies, or controlled comparisons with synthetic data, are needed to validate these mechanisms. The multiclass model offers clear operational benefits beyond accuracy. In field settings, co-infections are common, which makes a binary workflow cumbersome. Separate, disease-specific models require prior knowledge of which pathogens are present. They can also reintroduce manual pre-sorting, which undermines automation. The multiclass model provides a single, one-step output that quantifies both diseases simultaneously. This simplifies its use in breeding programs. Although both approaches achieved near-perfect genotype-level agreement with ground truth severity ($r > 0.99$; Fig. 5, Table 5), the multiclass model avoids the practical constraints of maintaining separate disease-specific pipelines. Overall, the multiclass model provides an effective balance between improved segmentation performance and a streamlined, scalable workflow. Importantly, the multiclass model provides actionable information at both the population and genotype levels. As shown in Section 3.4 and Supplementary Table S7, the model produces independent severity estimates for brown rust and ramularia, even when both diseases co-occur on the same leaves. This allows breeders to rank genotypes according to their relative susceptibility to each disease, rather than relying on composite or averaged measures. Predicted rankings closely match ground truth assessments, confirming that the model preserves biologically meaningful patterns across genotypes. At the population level, the model recovered expected contrasts between winter barley (brown rust-susceptible) and spring barley, and also captured temporal differences within the spring populations, as shown in Supplementary Figure S6 and Supplementary Tables S8–S9. Leaves collected in 2024, sampled slightly later in the season, exhibited high ramularia severity, while the 2025 collection, sampled earlier to capture the onset of pustule formation, showed substantially lower severity. The model accurately reflects this level of biological detail, distinguishing not only between populations but also between different disease stages within the same population, demonstrating its sensitivity and reliability for multi-disease phenotyping. By providing simultaneous, interpretable, and temporally resolved outputs across multiple diseases, the multiclass approach offers a practical, high-precision tool for high-throughput breeding programs.

### Limitations, error analysis, and future directions

Several limitations should be considered when interpreting these results. First, the pixel-level annotations, while generated with expert input, constitute a high-quality “silver standard” rather than a definitive gold standard. Inevitably, uncertainty at lesion boundaries and the visual similarity between disease symptoms and senescent or necrotic tissue introduce label noise. This is illustrated by the qualitative examples in Figs. 3 and 4, and is consistent with the genotype-level deviations observed in Fig. 5. Inspection of the genotype-level outliers suggested two distinct sources of error. For the brown rust outlier (Genotype 1 in Fig. 5A), the model segmented plausible lesions that were absent from the annotations in the ground truth, leading to an overestimation of diseased area percentage. In contrast, for the ramularia outlier (Genotype 2 in Fig. 5B), the model misclassified necrotic patterns as disease lesions, again increasing the estimated diseased area. Such cases likely reflect genuine visual ambiguity that also challenges human experts, and suggest that necrosis arising from biotic and abiotic causes remains difficult to disentangle from image appearance alone. The instance-level detection results provide additional insight into the remaining challenges (Table 4). While lesion localisation was strong at a lenient IoU threshold (F1@0.2: 0.77–0.78), performance dropped markedly under the stricter threshold requiring accurate boundary alignment (F1@0.5: 0.41–0.44). This pattern indicates that boundary precision, rather than lesion detection, is the main limitation. Given the small lesion size (see Supplementary Figure S2B), even minor contour discrepancies, whether due to model uncertainty or annotation inconsistency, can cause larger reductions in IoU. Beyond modelling, data acquisition is a practical bottleneck. The current workflow of leaf mounting and flatbed scanning produces high-quality images but is too slow for large-scale field phenotyping. Switching to mobile phone imaging or autonomous robots could increase throughput but might introduce additional variability in backgrounds, illumination, viewpoint, and zoom levels, which typically reduces effective resolution. This is particularly important for small lesions, which may be difficult to capture reliably. While the model is tailored to barley-specific symptoms, the segmentation framework is broadly applicable and could be adapted to other crops given suitable training data. Progress in this direction will depend on the availability of high-quality, pixel-level annotations and standardised imaging in other species, enabling systematic assessment of cross-species generalisability.

Therefore, future work should target both annotation efficiency and robustness under field conditions. Due to the high cost of expert pixel-level labels, it is important to explore weakly supervised and semi-supervised approaches that leverage unlabelled images, as well as methods designed to be robust to label noise. Additionally, real-world deployment requires moving beyond a closed set of known symptoms. Ideally, systems would segment known diseases, flag atypical or previously unseen symptoms, and distinguish disease-like necrosis from abiotic stress or senescence. Finally, architectures that capture broader context more effectively may reduce confusion among co-occurring symptoms and improve boundary delineation, particularly for small, irregular lesions. Together, these approaches could support the development of scalable phenotyping tools that can withstand the complexities of agricultural environments.

## Conclusion

This study evaluated whether a single multiclass deep learning model can identify and distinguish two visibly similar barley foliar diseases in field images. A multiclass U-Net with a ConvNeXt Tiny encoder jointly segmented brown rust and ramularia and outperforms disease-specific binary models for both classes, relatively improving Dice score by 47.5% for brown rust (0.59 vs. 0.40) and 13.2% for ramularia (0.60 vs. 0.53), while also increasing lesion-level recall.

The breeding-oriented phenotyping, both binary and multiclass approaches, produced near-identical genotype ordering, with genotype-level correlations exceeding $r > 0.99$ between predicted and annotated severity. However, binary models are operationally impractical under co-infection because they require separate disease-specific pipelines and reintroduce manual pre-sorting. Despite occasional overestimation under symptom ambiguity, the multiclass model provides a unified, scalable solution for multi-disease quantification in a single inference pass, supporting high-throughput resistance screening in breeding programs.

## Supplementary Information

Supplementary Material 1.

## Data Availability

The annotated dataset and the code implementing our machine learning–based model are publicly available on Mendeley Data (https://doi.org/10.17632/4ny92p2r8f.1) and GitHub (https://github.com/grimmlab/BarleyDiseaseSegmentation).

## References

[1] Communication from the Commission to the European Parliament, the Council, the European Economic and Social Committee and the Committee of the Regions - A Farm to Fork Strategy for a fair, healthy and environmentally-friendly food system. 20 May 2020.

[2] Hughes D, Salathé M. An open access repository of images on plant health to enable the development of mobile disease diagnostics. 2015. arXiv preprint arXiv:1511.08060.

[3] Cotterill P, Rees R, Platz G, Dill-Macky R. Effects of leaf rust on selected Australian barleys. Aust J Exp Agric. 1992;32(6):747–51.

[4] Park R, Golegaonkar P, Derevnina L, et al. Leaf rust of cultivated barley: pathology and control. Annu Rev Phytopathol. 2015;53:565–89. 26047566 10.1146/annurev-phyto-080614-120324

[5] Pinnschmidt HO, Jørgensen LN. Yield effects of ramularia leaf spot on spring barley. Asp Appl Biol. 2009;92:57–66.

[6] Xi K, Xue A, Burnett P, et al. Quantitative resistance of barley cultivars to Rhynchosporium secalis. Can J Plant Path. 2000;22(3):217–23.

[7] Baweja P, Kumar S, Kumar G. Fertilizers and pesticides: their impact on soil health and environment. In: Giri B, Varma A, editors. Soil biology. Berlin: Springer; 2020. p. 265–85.

[8] Thudi M, Palakurthi R, Schnable JC, Chitikineni A, Dreisigacker S, Mace E, et al. Genomic resources in plant breeding for sustainable agriculture. J Plant Physiol. 2021;257:153351. 33412425 10.1016/j.jplph.2020.153351 PMC7903322

[9] Fetch TG Jr, Steffenson BJ. Rating scales for assessing infection responses of barley infected with cochliobolus sativus. Plant Dis. 1999;83(3):213–7. 30845496 10.1094/PDIS.1999.83.3.213

[10] Saari,E. E. and Prescott,J. M., 19751634613, English, Journal article, 59, (5), Plant Disease Reporter, (377–380), A scale for appraising the foliar intensity of wheat diseases., (1975)

[11] EPPO (European and Mediterranean Plant Protection Organization). (2012). Foliar and ear diseases on cereals. EPPO Bulletin, 42(3), 419–425. 10.1111/epp.2613

[12] Pethybridge SJ, Nelson SC. Leaf doctor: a new portable application for quantifying plant disease severity. Plant Dis. 2015;99(10):1310–6. 30690990 10.1094/PDIS-03-15-0319-RE

[13] Ulrich M, Brain L, Zhang J, Gendall AR, Lück S, Douchkov D, et al. Foliar disease resistance phenomics of fungal pathogens: image-based approaches for mapping quantitative resistance in cereal germplasm. Theor Appl Genet. 2025;138(9):232. 40875020 10.1007/s00122-025-05017-4 PMC12394310

[14] Basak P, Kashyap N, Gurjar MS, Patidar R, Ramesh GV. Recent advances in spot blotch of barley and its future perspectives. Plant Pathol. 2025;74(9):2579–97.

[15] Pandey C, Sommer SG, Roitsch T, Schulz A. Phenotyping-based spectral signatures uncover barley cultivars’ sensitivity to combined mildew and drought treatment. Smart Agric Technol. 2025;11:101000.

[16] Barl L, Debastiani Benato B, Genze N, Grimm DG, Gigl M, Dawid C, et al. The combined effect of decreased stomatal density and aperture increases water use efficiency in maize. Sci Rep. 2025;15(1):13804. 40258909 10.1038/s41598-025-94833-1 PMC12012185

[17] Czymmek KJ, Duncan KE, Berg H. Realizing the full potential of advanced microscopy approaches for interrogating plant-microbe interactions. Mol Plant Microbe Interact. 2023;36(4):245–55. 36947723 10.1094/MPMI-10-22-0208-FI

[18] Genze N, Ajekwe R, Güreli Z, Haselbeck F, Grieb M, Grimm DG. Deep learning-based early weed segmentation using motion blurred uav images of sorghum fields. Comput Electron Agric. 2022;202:107388.

[19] Ronneberger O, Fischer P, Brox T. U-net: Convolutional networks for biomedical image segmentation. In: International Conference on Medical Image Computing and Computer-assisted Intervention. Springer. 2015;234–241.

[20] Dinesh P, Lakshmanan R. Multiclass semantic segmentation for prime disease detection with severity level identification in citrus plant leaves. Sci Rep. 2025;15(1):21208. 40596020 10.1038/s41598-025-04758-y PMC12217236

[21] Goncalves JP, Pinto FA, Queiroz DM, Villar FM, Barbedo JG, Del Ponte EM. Deep learning architectures for semantic segmentation and automatic estimation of severity of foliar symptoms caused by diseases or pests. Biosys Eng. 2021;210:129–42.

[22] Hai T, Shao Y, Zhang X, Yuan G, Jia R, Fu Z, et al. An efficient model for leafy vegetable disease detection and segmentation based on few-shot learning framework and prototype attention mechanism. Plants. 2025;14(5):760. 40094752 10.3390/plants14050760 PMC11902100

[23] Kaur P, Harnal S, Gautam V, Singh MP, Singh SP. Hybrid deep learning model for multi biotic lesions detection in solanum lycopersicum leaves. Multimed Tools Appl. 2024;83(3):7847–71.

[24] Polly R, Devi EA. Semantic segmentation for plant leaf disease classification and damage detection: a deep learning approach. Smart Agric Technol. 2024;9:100526.

[25] Upadhyay N, Gupta N. Seglearner: a segmentation based approach for predicting disease severity in infected leaves. Multimed Tools Appl. 2025. . 10.1007/s11042-025-20838-7

[26] Zhao C, Li C, Wang X, Wu X, Du Y, Chai H, et al. Plant disease segmentation networks for fast automatic severity estimation under natural field scenarios. Agriculture. 2025;15(6):583.

[27] Holan KL, White CH, Whitham SA. Application of a u-net neural network to the puccinia sorghi-maize pathosystem. Phytopathology®. 2024;114(5):990–9. 38281155 10.1094/PHYTO-09-23-0313-KC

[28] Arinicheva IV, Volkova GV, Yakhnik YV, Arinichev IV. Computer vision for monitoring and accounting pyrenophora teres of winter barley. News Kabardino-Balkarian Sci Cent Russ Acad Sci. 2024;26(2):72–9.

[29] Rezaei M, Diepeveen D, Laga H, Gupta S, Jones MG, Sohel F. A transformer-based few-shot learning pipeline for barley disease detection from field-collected imagery. Comput Electron Agric. 2025;229:109751.

[30] Zadoks JC, Chang TT, Konzak CF. A decimal code for the growth stages of cereals. Weed Res. 1974;14(6):415–21.

[31] Schilling MP, Scherr T, Muenke FR, Neumann O, Schutera M, Mikut R, et al. Automated annotator variability inspection for biomedical image segmentation. IEEE Access. 2022;10:2753–65.

[32] Zuiderveld KJ. Contrast limited adaptive histogram equalization. In: Graphics Gems. 1994. Accessed 22 January 2026. 10.1016/B978-0-12-336156-1.50061-6

[33] Zhang Y, Shi N, Zhang H, Zhang J, Fan X, Suo X. Appearance quality classification method of Huangguan pear under complex background based on instance segmentation and semantic segmentation. Front Plant Sci. 2022;13:914829. 36340375 10.3389/fpls.2022.914829 PMC9627623

[34] Bernett J, Blumenthal DB, Grimm DG, Haselbeck F, Joeres R, Kalinina OV, et al. Guiding questions to avoid data leakage in biological machine learning applications. Nat Methods. 2024;21(8):1444–53. 39122953 10.1038/s41592-024-02362-y

[35] Hernández I, Silva R, Melo-Pinto P, Gutiérrez S, Tardaguila J. Early detection of downy mildew in vineyards using deep neural networks for semantic segmentation. Biosys Eng. 2025;252:15–31.

[36] Li Y, Qiao T, Leng W, Jiao W, Luo J, Lv Y, et al. Semantic segmentation of wheat stripe rust images using deep learning. Agronomy. 2022;12(12):2933.

[37] Pandiri DK, Murugan R, Goel T. Arm-unet: attention residual path modified unet model to segment the fungal pathogen diseases in potato leaves. SIViP. 2025;19(1):80.

[38] Azad R, Aghdam EK, Rauland A, Jia Y, Avval AH, Bozorgpour A, et al. Medical image segmentation review: the success of u-net. IEEE Trans Pattern Anal Mach Intell. 2024. . 10.1109/TPAMI.2024.3435571 39167505 10.1109/TPAMI.2024.3435571

[39] He K, Zhang X, Ren S, Sun J. Deep residual learning for image recognition. 2015. arXiv preprint arXiv:1512.03385

[40] Wightman R. PyTorch image models. GitHub. 2019. 10.5281/zenodo.4414861.

[41] Tan M, Le Q. Efficientnet: Rethinking model scaling for convolutional neural networks. In: International Conference on Machine Learning. PMLR. 2019;6105–6114.

[42] Liu Z, Mao H, Wu C-Y, Feichtenhofer C, Darrell T, Xie S. A convnet for the 2020s. In: Proceedings of the IEEE/CVF Conference on Computer Vision and Pattern Recognition. 2022;11976–11986.

[43] Bergstra J, Yamins D, Cox D. Making a science of model search: Hyperparameter optimization in hundreds of dimensions for vision architectures. In: International Conference on Machine Learning. PMLR. 2013;115–123.

[44] Azad R, Heidary M, Yilmaz K, Hüttemann M, Karimijafarbigloo S, Wu Y, Schmeink A, Merhof D. Loss functions in the era of semantic segmentation: A survey and outlook. 2023. arXiv preprint arXiv:2312.05391

[45] Loshchilov I, Hutter F. Decoupled weight decay regularization. 2017. arXiv preprint arXiv:1711.05101

[46] Müller D, Soto-Rey I, Kramer F. Towards a guideline for evaluation metrics in medical image segmentation. BMC Res Notes. 2022;15(1):210. 35725483 10.1186/s13104-022-06096-y PMC9208116

