# Supplementary material for: Deep learning–based identification of visually similar foliar diseases in field-grown barley
Source: Plant Methods. 2026 Apr 18;22:41. doi: 10.1186/s13007-026-01532-7 (PMC13097865; doi:10.1186/s13007-026-01532-7)
Supplement: Supplementary file 1 — Supplementary Material 1. [file 13007_2026_1532_MOESM1_ESM.pdf]

Supplementary Information for Deep Learning–Based Identification of Visually Similar Foliar Diseases in Field-Grown Barley

Sofia Martello[1,2], Nikita Genze[1,2], Dominik G. Grimm[1,2,3*]

[1]Technical University of Munich, Campus Straubing for Biotechnology and Sustainability, Bioinformatics, 94315 Straubing, Germany.
[2]Weihenstephan-Triesdorf University of Applied Sciences, Bioinformatics, 94315 Straubing, Germany.
[3]Technical University of Munich, TUM School of Computation, Information and Technology, 85748, Garching, Germany.

*Corresponding author(s). E-mail(s): dominik.grimm@tum.de; Contributing authors: sofia.martello@tum.de; nikita.genze@tum.de;

1 Supplementary Notes

1.1 Note S1: Detailed Evaluation Metrics

The full mathematical definitions and justifications for all evaluation metrics used in the main study are provided here. A suite of metrics was employed to evaluate different aspects of segmentation and detection performance. The choice of metric aggregation, i.e. the calculation of the mean Dice score for all classes or for a single class, was tailored to the specific objective of each experimental phase. During the HPO and encoder selection phase, the objective was to compare different architectures on the same task. Therefore, global metrics, specifically the mean Dice score (mDice) and mean Intersection over Union (mIoU), were used as they enabled the identification of which model achieved the best score across all classes for a given task. This provided a single, comparable score for ranking encoder-task combinations. For the final inference and model comparison phase, the goal was to evaluate the binary versus multiclass modelling approaches. To compare these two distinct strategies, semantic segmentation metrics needed to be computed per class, as using binary metrics on multiclass tasks distorts performance evaluations Müller et al. [1]. The per-class evaluation allowed

a direct comparison between, for instance, the ramularia segmentation performance of the dedicated binary model and the ramularia class output from the multiclass model, both evaluated against the same ramularia ground truth. Model performance was evaluated on a held-out test set comprising 59 leaves from 10 distinct genotypes.

The evaluation metrics are now described in detail. Precision and recall were analysed at each stage. Precision measures the model's false positive rate, while recall measures its sensitivity to missed lesions. The primary metric for optimisation and model selection was the Dice score, which measures pixel-wise overlap between predicted and ground truth masks:

$$\text{Dice} = \frac{2 \times TP}{2 \times TP + FP + FN}. \quad (1)$$

It was chosen for its robustness to class imbalance and its ability to balance precision and recall, making it particularly suitable for segmenting small, rare lesions [2]. To provide a stricter assessment of segmentation quality, the Intersection over Union (IoU) was also reported:

$$\text{IoU} = \frac{TP}{TP + FP + FN}. \quad (2)$$

This metric penalises poor localisation more heavily than the Dice score and offers a straightforward interpretation of area-based accuracy [3].

Beyond pixel-level segmentation, instance-level detection metrics were crucial for evaluating the model's ability to identify individual lesions:

$$\text{Precision}_{\text{det}} = \frac{|\{\text{detections with IoU} > \tau\}|}{|\{\text{all detections}\}|}, \quad (3)$$

$$\text{Recall}_{\text{det}} = \frac{|\{\text{ground truths with IoU} > \tau\}|}{|\{\text{all ground truths}\}|}, \quad (4)$$

$$\text{Detection } F1@\tau = \frac{2 \cdot \text{Precision}_{\text{det}} \cdot \text{Recall}_{\text{det}}}{\text{Precision}_{\text{det}} + \text{Recall}_{\text{det}}}. \quad (5)$$

where τ is an IoU threshold used to determine whether a predicted detection matches a ground truth annotation.

After analysing detection F1 scores at 10 thresholds, as shown in Supplementary Figure 1, two thresholds were selected for further investigation. F1 with a threshold of 0.2 measures the model's capability to locate lesions, even with imperfect boundary delineation; a true positive is counted if the predicted and ground-truth instance have an overlap greater than 20%. Conversely, F1 with a threshold of 0.5 evaluates precise lesion localisation and boundary accuracy, with a true positive requiring 50% overlap.

To assess practical utility for plant breeding applications, the model's predictions were evaluated for their ability to quantify disease severity at the genotype level. This was done by correlating the disease-area percentages derived from model predictions

with those from ground-truth annotations. Pearson correlation coefficients were used:

$$r = \frac{\sum(x_i - \bar{x})(y_i - \bar{y})}{\sqrt{\sum(x_i - \bar{x})^2 \cdot \sum(y_i - \bar{y})^2}}, \tag{6}$$

where x_i represents ground truth disease area percentage values, y_i represents predicted disease area percentage values, $\bar{x}$ and $\bar{y}$ are their respective means, n is the number of samples, and $r \in [-1, +1]$ is the correlation coefficient.

The correlation between model-predicted and ground-truth disease areas was calculated at two levels. First, the leaf-level correlation (r_{leaf}) was calculated across all individual leaves in the test set, measuring the model's accuracy for single-leaf assessment. Second, the genotype-level correlation (r_{genotype}) was calculated using genotype means, with the predicted and ground-truth disease areas averaged across all leaves within each genotype. This metric directly evaluates the model's ability to rank genotypes by disease severity, which is the primary requirement for breeding programmes:

$$r_{\text{leaf}} = \text{corr}(\bar{x}_i, \bar{y}i), \;\; r\text{genotype} = \text{corr}(\bar{x}_g, \bar{y}_g). \tag{7}$$

To complement correlation metrics, error-based measures were computed at the genotype level. Both MAE and RMSE are expressed on a 0–100 % scale, consistent with the range of disease severity values.

The mean absolute error (MAE) is defined as

$$\text{MAE} = \frac{1}{n} \sum_{i=1}^{n} |y_i - \hat{y}_i| \, , \tag{8}$$

where y_i is the ground-truth disease severity (%) and $\hat{y}_i$ is the predicted value for genotype i, and n is the number of genotypes. MAE provides a simple measure of average prediction error that reflects typical deviations without placing undue weight on large errors.

The root mean square error (RMSE) is defined as

$$\text{RMSE} = \sqrt{\frac{1}{n} \sum_{i=1}^{n} (y_i - \hat{y}_i)^2}. \tag{9}$$

RMSE penalises larger deviations more heavily than MAE, making it sensitive to extreme prediction errors. Together, MAE and RMSE provide complementary absolute measures of model prediction accuracy on the same 0–100 % scale as the disease severity, allowing direct interpretation of agreement with ground-truth disease areas.

All metrics for the final inference were calculated per leaf and averaged across the test set, except for genotype-level correlations, which were computed across genotype-level averages.

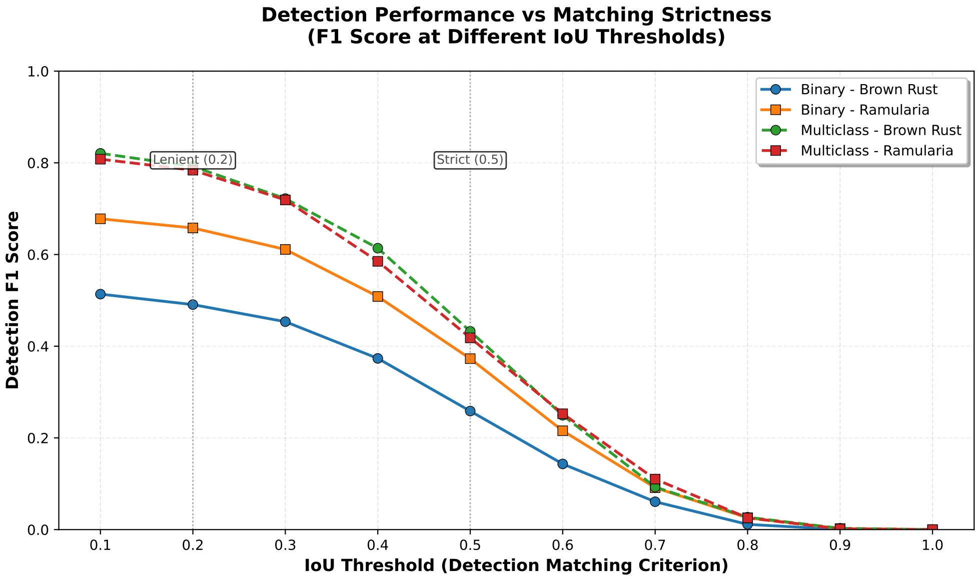

Fig. 1: Supplementary Figure S1: Detection F1 scores across 10 IoU thresholds. Thresholds 0.2 and 0.5 were selected for detailed analysis.

1.2 Note S2: Quantitative Data Characterisation

This note provides the quantitative analysis supporting the dataset description in the main text. Brown rust lesions were significantly brighter, while ramularia lesions were characterised by a higher Red-to-Green (R/G) ratio, primarily due to a suppression of green reflectance (Supplementary Figure 2A). However, a projection into a 2D colour space (Supplementary Figure 2B) shows a clear overlap between the two classes, indicating that colour alone is insufficient for separation. Ramularia lesions were found to be significantly larger on average and more variable in size than the typically smaller, more uniform brown rust lesions, as shown in Supplementary Figure 2C.

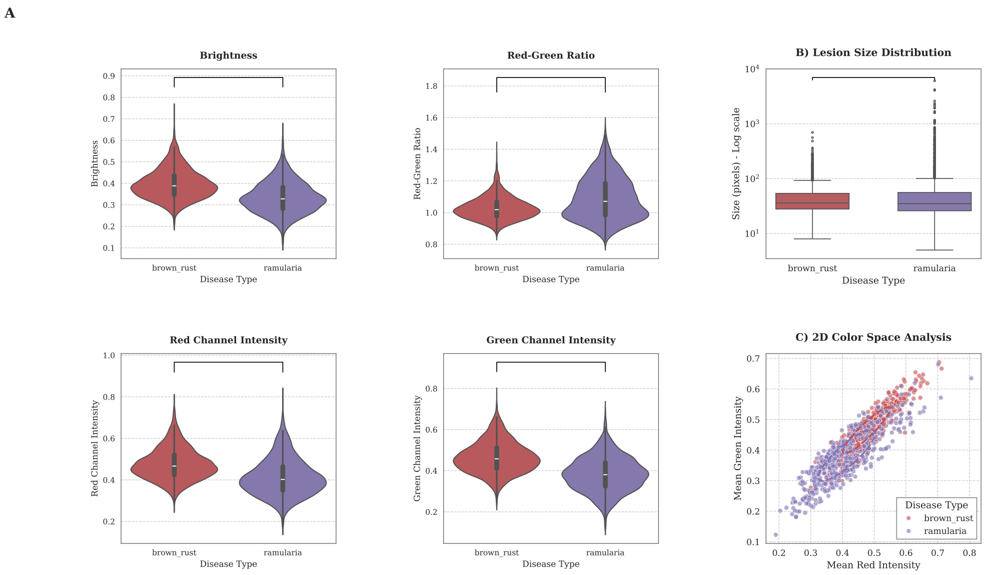

Fig. 2: Supplementary Figure S2: Data characterisation analysis showing visual and statistical properties of co-infecting barley diseases. (A) Violin plots comparing brightness, R/G ratio, and colour intensity. (B) Projection into the Red-Green colour space demonstrates substantial overlap. (C) Size distribution shows strong skew, with ramularia lesions being larger and more variable than brown rust lesions.

1.3 Note S3: Loss Function Formulation

The combined Dice and Focal loss used for training is defined as:

$$\mathcal{L}_{\text{FocalDice}} = \lambda \mathcal{L}_{\text{Dice}} + (1 - \lambda)\mathcal{L}_{\text{Focal}} \quad (10)$$

where:

- $\mathcal{L}_{\text{Dice}}$: weighted Dice loss (micro-averaged)
- $\mathcal{L}_{\text{Focal}}$: Focal loss with class weights $w_c \propto 1/\text{freq}_c$
- λ: optimised hyperparameter balancing the two components
- Background pixels were excluded from loss calculation

2 Supplementary Tables

Table 1: Supplementary Table S1: Summary of leaf sample collection by disease population. Samples were collected from field trials across three locations in Germany (Irlbach, Paitzkofen) and France (Estrées Saint Denis) during the 2024 and 2025 growing seasons. The brown rust population represents winter barley, while the ramularia population represents spring barley.

Disease Population	Collection Year	Location	Number of Genotypes	Total Leaves
ramularia	2024	Irlbach(DE)	201	804
	2025	Paitzkofen(DE)	204	816
brown rust	2024	Estrees Saint Denis(F)	238	2856

Table 2: Supplementary Table S2: Dataset composition details across training, validation, and test splits. The dataset exhibits severe class imbalance, with healthy tissue accounting for the majority of pixel counts. The lower section shows aggregate disease totals: brown rust lesions are more numerous but have a smaller total pixel area than ramularia lesions.

	Train	Validation	Test
Leaves	242	35	59
Disease Lesions	147,025	14,490	4811
Disease Pixels	10,964,546	1,192,665	298,637
Disease totals across all sets:			
	Brown rust		Ramularia
Lesions	106,330		59,996
Pixels	5,035,874		7,419,974

Table 3: Supplementary Table S3: Data augmentation pipeline configuration applied to training images. All augmentations were applied sequentially with independent probabilities during training to increase dataset variability and improve model robustness. The RandomSizedCrop operation crops random portions of the image and resizes them back to the original 512×512 dimensions.

**Augmentation**	**Description**	**Probability**
HorizontalFlip	Flip image horizontally (left-right)	50%
VerticalFlip	Flip image vertically (top-bottom)	50%
RandomRotate90	Randomly rotate image by 90°, 180°, or 270°	75%
Transpose	Swap image axes (similar to 90° rotation)	50%
RandomSizedCrop	Crop random portion and resize to 512×512	25%

Table 4: Supplementary Table S4: Hyperparameter search spaces for Bayesian optimisation. Two-phase search strategy: initial broad search across all parameters, followed by refined search with some parameters fixed at optimal values identified in phase one (indicated by "Fixed:"). Parameters above the horizontal line were optimised in both phases; those below were fixed during the refined search.

Parameter	Description	Initial Search Space	Refined Search Space
lr	Learning rate	$[1 \times 10^{-5}, 1 \times 10^{-2}]$	$[1 \times 10^{-6}, 1 \times 10^{-2}]$
weight_decay	L2 regularization weight	$[1 \times 10^{-6}, 1 \times 10^{-2}]$	$[1 \times 10^{-8}, 1 \times 10^{-1}]$
decoder_dropout	Dropout rate in decoder layers	$[0.1, 0.3]$	$[0.05, 0.25]$
bottleneck_dropout	Dropout rate in bottleneck layer	$[0.1, 0.5]$	**Fixed:** 0.385
dice_weight	Weight for Dice loss component	$[0.7, 1.0]$	**Fixed:** 0.721
focal_alpha	Alpha parameter for focal loss	$[0.6, 0.8]$	**Fixed:** 0.737
focal_gamma	Gamma parameter for focal loss	$[1.5, 2.8]$	**Fixed:** 1.847

Table 5: Supplementary Table S5: Visual assessment scale for disease severity scoring used by pathologists. The 9-point logarithmic scale converts percentage disease area to ordinal scores for ranking genotype resistance. Ranges are non-linear to match human perception of disease severity, with finer discrimination at lower severity levels relevant for breeding selection.

Score	Disease Area (%)	Description
1	0.0	No symptoms
2	0.0–2.0	Trace infection
3	2.0–5.0	Slight infection
4	5.0–8.0	Light infection
5	8.0–14.0	Moderate infection
6	14.0–22.0	Considerable infection
7	22.0–37.0	Severe infection
8	37.0–61.0	Very severe infection
9	61.0–100.0	Extremely severe infection

Table 6: Supplementary Table S6: Encoder architecture comparison on validation set. Mean Dice scores (mDice) are computed differently for binary (2-class: background + disease) versus multiclass (3-class: background + brown rust + ramularia) tasks and are not directly comparable across columns. ConvNeXt Tiny achieved the highest performance for all three task formulations and was selected for final model training.

Encoder	**Binary Ramularia** mDice (2-class)	**Binary Brown Rust** mDice (2-class)	**Multiclass** mDice (3-class)
ResNet34	0.7726	0.6972	0.6488
EfficientNet	0.7607	0.7095	0.6320
ConvnextTiny	**0.7771**	**0.7228**	**0.6595**

Table 7: Supplementary Table S7: Brown rust and ramularia percentage measurements and derived visual scores by genotype across all model formulations. Percentage values represent disease severity estimates, while scores are visual assessment values (1-9 scale) derived from percentage measurements and mapped to the standard visual assessment table. Prediction errors (deviations of exactly one score level) are highlighted in bold. The binary rust model only predicts brown rust, the binary ramularia model only predicts ramularia, and the multiclass model predicts both diseases simultaneously.

Genotype	Rust (%)			Rust Score			Ram (%)			Ram Score		
	Multi	Bin	GT	Multi	Bin	GT	Multi	Bin	GT	Multi	Bin	GT
9635	5.04[a]	4.26	4.15	3[a]	3	3	0.34	0.42	0.29	2	2	2
694037	0.25	0.54	0.21	2	2	2	2.29	1.74	1.12	**3**	2	2
694044	0.15	0.20	0.10	2	2	2	0.96	0.74	0.80	2	2	2
694040	0.26	0.41	0.21	2	2	2	0.66	0.37	0.47	2	2	2
694038	0.24	0.44	0.16	2	2	2	1.09	0.77	0.65	2	2	2
694042	0.48	0.76	0.49	2	2	2	1.88	1.14	1.34	2	2	2
694041	0.25	0.38	0.20	2	2	2	1.78	0.93	0.92	2	2	2
694043	0.32	0.75	0.21	2	2	2	1.97	1.26	1.55	2	2	2
41561	3.22	2.90	1.39	**3**	**3**	2	11.52	13.20	12.59	5	5	5
694039	0.10	0.29	0.08	2	2	2	1.39	0.98	1.09	2	2	2

[a]The predicted brown rust percentage from the multiclass model (5.04%) is 0.04% above the threshold for score 3; however, given the minimal deviation, it was retained as score 3 rather than rounded up to score 4. Bold values indicate prediction errors (deviation of exactly one point on the visual assessment scale). Multi = Multiclass model, Binary = Binary disease-specific model, GT = Ground truth.

Table 8: Supplementary Table S8: Population-level disease severity in the test set. Winter barley shows significantly higher brown rust severity compared to both spring populations, while spring barley shows significantly higher ramularia severity. Statistical comparisons: Winter vs Spring 2024 (brown rust: $p = 0.026$, ramularia: $p = 0.013$), Winter vs Spring 2025 (brown rust: $p < 0.001$, ramularia: $p < 0.001$), Mann-Whitney U test, one-sided.

Population	n (leaves)	Brown Rust (%)		Ramularia (%)	
		Predicted	Ground Truth	Predicted	Ground Truth
Winter	11	5.04 ± 3.21	4.15 ± 2.89	0.34 ± 0.28	0.29 ± 0.24
Spring 2024	2	3.22 ± 1.45	1.39 ± 0.92	11.52 ± 4.67	12.59 ± 5.12
Spring 2025	46	0.26 ± 0.18	0.21 ± 0.15	1.54 ± 1.02	1.01 ± 0.78

Table 9: Supplementary Table S9: Population-level disease severity in the large unlabelled dataset. The expected biological patterns are statistically confirmed for Winter vs Spring 2024 comparisons (Mann-Whitney U test, one-sided, $p < 0.001$ for both diseases). Note the unexpectedly low ramularia severity in Spring 2025 population.

**Population**	**n (leaves)**	**Brown Rust (%)**	**Ramularia (%)**
Winter	2,092	2.90 ± 1.89	1.99 ± 1.34
Spring 2024	669	1.27 ± 0.98	23.00 ± 12.45
Spring 2025	882	0.25 ± 0.19	1.81 ± 1.23

Table 10: Supplementary Table S10: Mean absolute error (MAE) and root mean square error (RMSE) between predicted and ground truth disease severity (%) at the genotype level.

**Disease**	**MAE (%)**	**RMSE (%)**
Brown rust	0.31	0.64
Ramularia	0.52	0.64

3 Supplementary Figures

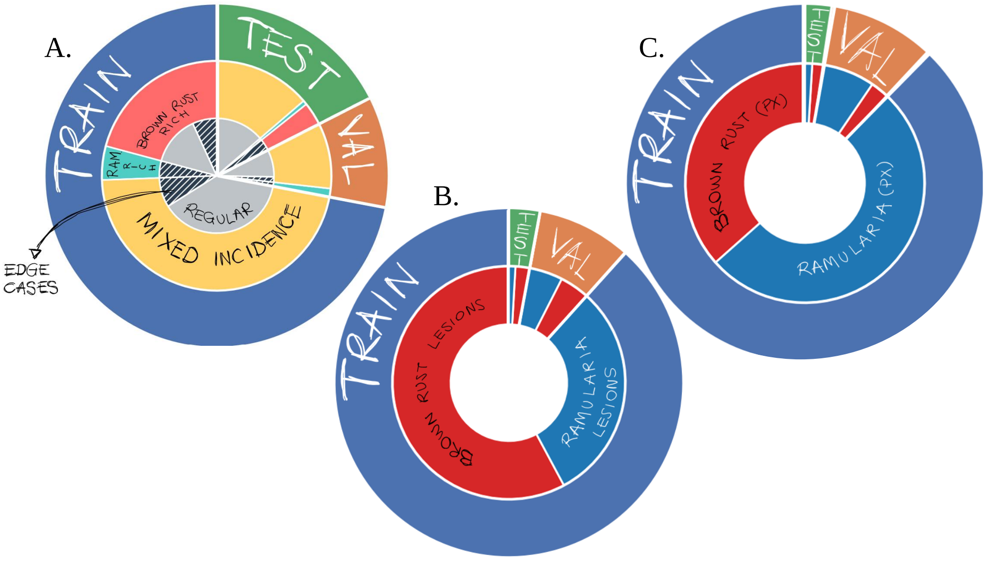

Fig. 3: Supplementary Figure S3: Multi-scale analysis of barley disease dataset composition and distribution. (A) Dataset composition across splits. (B) Lesion-level disease distribution. (C) Pixel-level disease distribution. All charts follow a ring structure where the outer ring shows the distribution across dataset splits (Train/Validation/Test), and the middle ring displays the composition within each split. Refer to Supplementary Table S2 for numerical details.

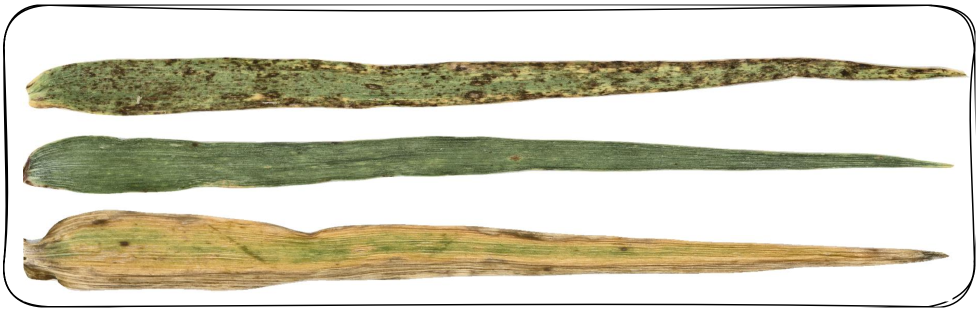

Fig. 4: Supplementary Figure S4: Example leaves showing co-infection of brown rust and ramularia, illustrating the visual similarity and complexity of the segmentation task. Both pathogens produce small, necrotic lesions with overlapping visual characteristics.

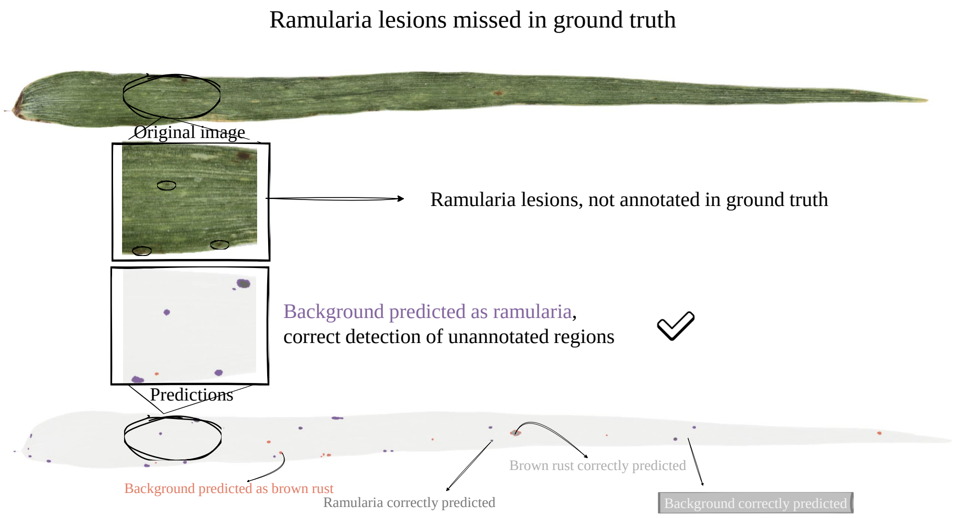

Fig. 5: Supplementary Figure S5: Example of the ground truth annotations being less accurate than the model predictions, in a lower disease severity leaf sample.

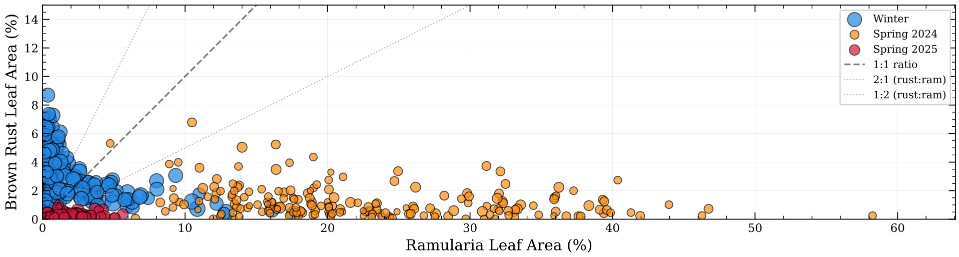

Fig. 6: Supplementary Figure S6: Population and temporal disease patterns. Scatter plot showing brown rust severity (y-axis) versus ramularia severity (x-axis) for individual leaves in the unlabelled dataset. Winter barley genotypes cluster in the brown rust-dominant region (high rust, low ramularia). Spring 2024 genotypes cluster in the ramularia-dominant region (low rust, high ramularia), being collected during the peak ramularia infection period. Spring 2025 genotypes collected earlier in the season show lower disease severity for both diseases.

References

[1] Müller, D., Soto-Rey, I., Kramer, F.: Towards a guideline for evaluation metrics in medical image segmentation. BMC Research Notes **15**(1), 210 (2022)

[2] Azad, R., Heidary, M., Yilmaz, K., Hüttemann, M., Karimijafarbigloo, S., Wu, Y., Schmeink, A., Merhof, D.: Loss functions in the era of semantic segmentation: A survey and outlook. arXiv preprint arXiv:2312.05391 (2023)

[3] Rahman, M.A., Wang, Y.: Optimizing intersection-over-union in deep neural networks for image segmentation. In: International Symposium on Visual Computing,

pp. 234–244 (2016). Springer
